# Supplementary material for: Heart Rate Variability in Subjects with Severe Allergic Background Undergoing COVID-19 Vaccination
Source: Vaccines (Basel). 2023 Mar 1;11(3):567. doi: 10.3390/vaccines11030567 (PMC10051914; doi:10.3390/vaccines11030567)
Supplement: Supplementary file 1 [file vaccines-11-00567-s001.zip › vaccines-2185344-supplementary.pdf]

Supplemental S1 Example of Kubios HRV data extraction

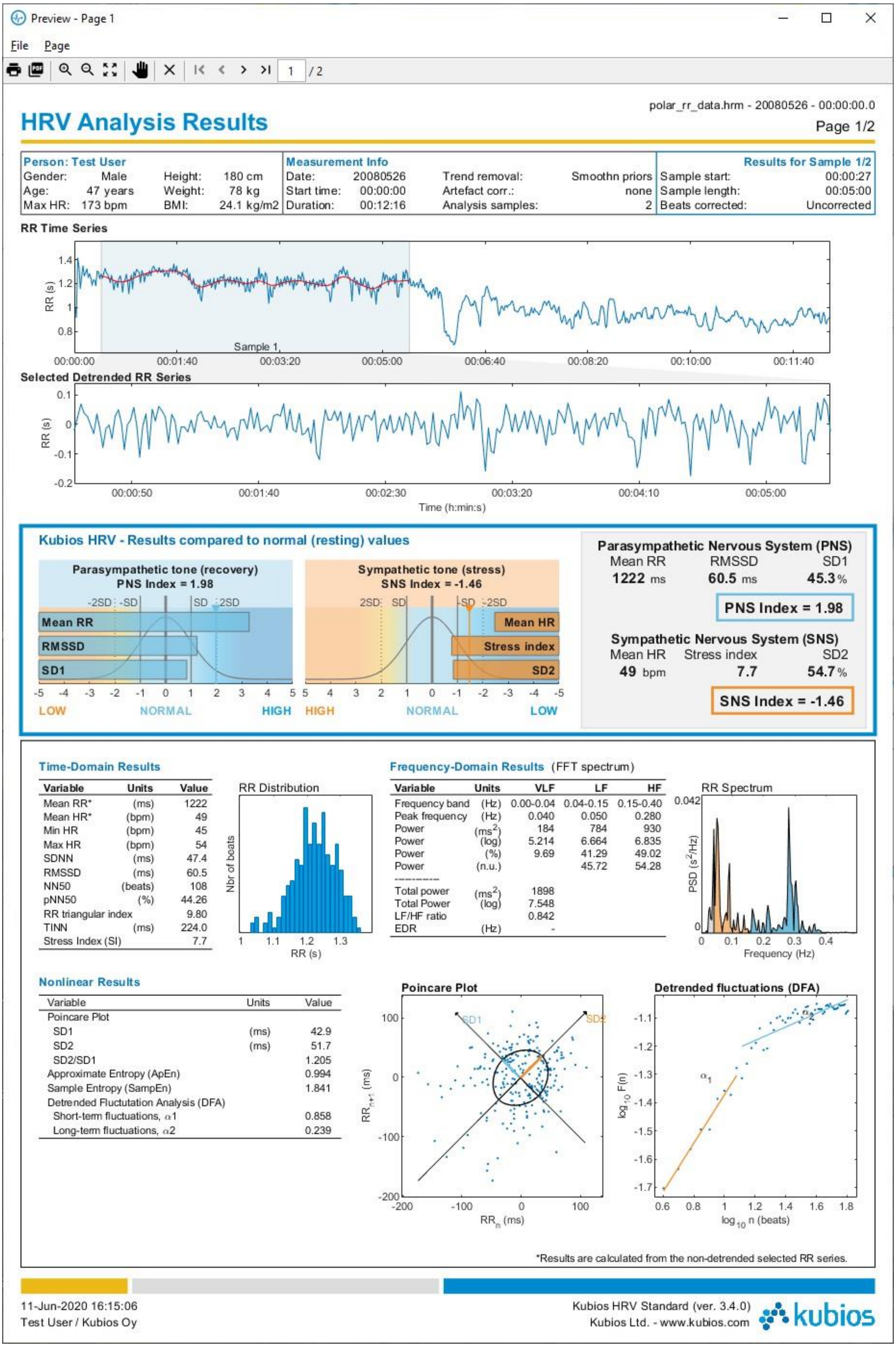

Preview - Page 1

File Page

1 / 2

polar_rr_data.hrm - 20080526 - 00:00:00.0

Page 1/2

HRV Analysis Results

**Person: Test User**				**Measurement Info**				**Results for Sample 1/2**	
Gender:	Male	Height:	180 cm	Date:	20080526	Trend removal:	Smoothn priors	Sample start:	00:00:27
Age:	47 years	Weight:	78 kg	Start time:	00:00:00	Artefact corr.:	none	Sample length:	00:05:00
Max HR:	173 bpm	BMI:	24.1 kg/m2	Duration:	00:12:16	Analysis samples:	2	Beats corrected:	Uncorrected

RR Time Series

Selected Detrended RR Series

Kubios HRV - Results compared to normal (resting) values

Parasympathetic tone (recovery)
PNS Index = 1.98

Sympathetic tone (stress)
SNS Index = -1.46

Parasympathetic Nervous System (PNS)

Mean RR	RMSSD	SD1
1222 ms	60.5 ms	45.3 %

PNS Index = 1.98

Sympathetic Nervous System (SNS)

Mean HR	Stress index	SD2
49 bpm	7.7	54.7 %

SNS Index = -1.46

Time-Domain Results

Variable	Units	Value
Mean RR*	(ms)	1222
Mean HR*	(bpm)	49
Min HR	(bpm)	45
Max HR	(bpm)	54
SDNN	(ms)	47.4
RMSSD	(ms)	60.5
NN50	(beats)	108
pNN50	(%)	44.26
RR triangular index		9.80
TINN	(ms)	224.0
Stress Index (SI)		7.7

Frequency-Domain Results (FFT spectrum)

Variable	Units	VLF	LF	HF
Frequency band	(Hz)	0.00-0.04	0.04-0.15	0.15-0.40
Peak frequency	(Hz)	0.040	0.050	0.280
Power	(ms^2)	184	784	930
Power	(log)	5.214	6.664	6.835
Power	(%)	9.69	41.29	49.02
Power	(n.u.)		45.72	54.28
Total power	(ms^2)	1898		
Total Power	(log)	7.548		
LF/HF ratio		0.842		
EDR	(Hz)	-		

Nonlinear Results

Variable	Units	Value
Poincare Plot		
SD1	(ms)	42.9
SD2	(ms)	51.7
SD2/SD1		1.205
Approximate Entropy (ApEn)		0.994
Sample Entropy (SampEn)		1.841
Detrended Fluctuation Analysis (DFA)		
Short-term fluctuations, $\alpha 1$		0.858
Long-term fluctuations, $\alpha 2$		0.239

Poincare Plot

Detrended fluctuations (DFA)

*Results are calculated from the non-detrended selected RR series.

11-Jun-2020 16:15:06
Test User / Kubios Oy

Kubios HRV Standard (ver. 3.4.0)
Kubios Ltd. - www.kubios.com

Supplemental S2 Allergy questionnaire

Questionnaire

Age --------------------- **Sex:** F □ M □

1-Have you ever had allergic reactions to drugs/vaccination? yes □ no □

2-Have you ever had allergic reactions to more than two classes of drugs? yes □ no □

3- Have you ever had allergic reactions to food? yes □ no □

Allergic Comorbidities

Rhinitis/Conjunctivitis yes □ no □

Asthma yes □ no □

Atopic Dermatitis yes □ no □

Chronic Itch yes□ no □

Contact Dermatitis yes □ no □

Chronic Urticaria yes □ no □

Reactions to Hymenoptera yes □ no □

Positive allergic tests (prick tests/IgE dosage) yes □ no □

Antiallergic therapies:

Antihistamine drugs yes □ no □

Montelukast yes □ no □

Steroids yes□ no □

General comorbidities (Mood disorders included)

Yes □ NO □

Specify with medications:

____

____

Covid-19 vaccine

First dose □ Second dose □ Third dose □

Have you ever had reactions to Covid-19 vaccination? yes □ no □

Type of reaction

____

____

How do you feel to have your vaccination in exclusive session?

More nervous □ | Same status □ | More relaxed □
